# Potential adjustment methodology for missing data and reporting delay in the HIV Surveillance System, European Union/European Economic Area, 2015

**DOI:** 10.2807/1560-7917.ES.2018.23.23.1700359

**Published:** 2018-06-07

**Authors:** Magdalena Rosinska, Nikos Pantazis, Janusz Janiec, Anastasia Pharris, Andrew J Amato-Gauci, Chantal Quinten, ECDC HIV/AIDS Surveillance Network

**Affiliations:** 1 National Institute of Public Health – National Institute of Hygiene, Warsaw, Poland; 2 National and Kapodistrian University of Athens, Athens, Greece; 3 European Centre for Disease Prevention and Control (ECDC), Stockholm, Sweden; 4 Members of the ECDC HIV/AIDS Surveillance Network are listed at the end of the article

**Keywords:** HIV infection, surveillance, missing values, reporting delay, multiple imputations, Europe

## Abstract

Accurate case-based surveillance data remain the key data source for estimating HIV burden and monitoring prevention efforts in Europe. We carried out a literature review and exploratory analysis of surveillance data regarding two crucial issues affecting European surveillance for HIV: missing data and reporting delay. Initial screening showed substantial variability of these data issues, both in time and across countries. In terms of missing data, the CD4^+^ cell count is the most problematic variable because of the high proportion of missing values. In 20 of 31 countries of the European Union/European Economic Area (EU/EEA), CD4^+^ counts are systematically missing for all or some years. One of the key challenges related to reporting delays is that countries undertake specific one-off actions in effort to capture previously unreported cases, and that these cases are subsequently reported with excessive delays. Slightly different underlying assumptions and effectively different models may be required for individual countries to adjust for missing data and reporting delays. However, using a similar methodology is recommended to foster harmonisation and to improve the accuracy and usability of HIV surveillance data at national and EU/EEA levels.

## Introduction

HIV remains one of the most important public health concerns in the European Union and European Economic Area (EU/EEA). Accurate data are therefore crucial to appropriately direct and evaluate public health response.

The European Centre for Disease Prevention and Control (ECDC) and the World Health Organization Regional Office for Europe (WHO/Europe) have jointly coordinated enhanced HIV/AIDS surveillance in the European Region since 2008. The general objectives of the surveillance system in EU/EEA countries include monitoring of trends over time and across countries. Specific HIV-related objectives include the monitoring of testing patterns, late HIV diagnoses, defined by low CD4^+^ counts (<350 cells/mm^3^), and mortality, as well estimating HIV incidence and prevalence stratified by key populations, e.g. transmission category and migrant status [1].

To meet these objectives, the long-term strategy states that improving the quality of surveillance data is needed [2]. Achieving this in practice poses challenges, especially given the heterogeneous national surveillance systems in the EU/EEA and that the routinely collected data are known to suffer from important quality limitations. The limitations originating from national data collection systems may include under-reporting or duplication of cases, delays in reporting, incompleteness of data and misclassification. Accounting for some of these limitations (e.g. assessment of under-reporting) requires additional data such as cohort studies or registries, while other issues, such as incompleteness and reporting delay, may be addressed directly within the surveillance datasets.

Missing data are a well-recognised problem within surveillance systems. When values for some variables are missing and cases with missing values are excluded from analysis, it may lead to biased and potentially less precise estimates [3,4]. In principle, whenever there are missing data or reporting delays, the accuracy of epidemiological distributions and trends should be interpreted with caution.

Reporting delay, the time from case diagnosis to notification, can lead to problems when analysing the most recent years, given that the information on some cases or variables may not have been collected yet because of national reporting process characteristics. This phenomenon is common in disease surveillance and also applies to HIV [5-8]. Rough adjustments for reporting delay were already implemented in the past in Europe [8,9], but further refinement of the existing applied methodology is needed to address this issue across more countries’ data.

The main purpose of this paper is to explore the issues of missing data and reporting delay in EU/EEA HIV surveillance data. We aim to quantify the extent to which these problems are present and to identify specific data characteristics that are relevant for data adjustments. Taking these characteristics into account, we also propose methods to adjust for missing data and reporting delay based on literature and existing national practices in EU/EEA countries.

## Methods

The analysis was based on HIV surveillance data from 31 EU/EEA countries uploaded to the European Surveillance System (TESSy) database by September 2015 [10].

All countries, apart from Liechtenstein, described their surveillance system as comprehensive. In six countries reporting was voluntary, whereas in the others it was mandatory [9]. In four countries (Bulgaria, Estonia, Ireland and Italy) historical data covering different periods before 2010 were reported in aggregate format (n = 23,131 cases). Full national coverage was achieved in 2012 in Italy and in 2013 in Spain; France started HIV surveillance in 2003. All cases reported in case-based format from 1980 or from the beginning of national surveillance up through 2014 were included in the analysis (n = 535,434 cases).

Descriptive analysis of missing values and reporting delay in the surveillance data was performed. In terms of missing values, the analysis focused on more recent data. For our analysis, this was defined as cases diagnosed in 2000 or later. It should be noted that some important variables, such as viral load, were excluded from the missing values analysis as they were only introduced with the 2015 revised reporting format and this format is not used by a percentage of the countries in the analysed dataset [9].

Historically, besides the year, only the quarter of the year of diagnosis and the quarter of notification were reported, and still the precise dates are not available for many countries. Moreover, data are uploaded to TESSy with a lag of two quarters, which makes short delays less relevant. Taking this into account, we defined reporting delay as the difference between the quarter when the case was diagnosed and the quarter when the case was reported. A nonparametric K-sample test on the equality of medians was used to compare the lengths of reporting delays across groups.

A consultation among the EU/EEA countries, in which 26 of 31 countries participated, was performed to identify methods already in use. In addition, we performed a scoping review to identify further available techniques, focusing on papers that reported on the surveillance applications. The scoping review included PubMed and Scopus database searches and a Google search using the search terms ‘missing data’/‘missing values’ or ‘reporting delay’. We retrieved literature published between 1985 and 2016 (Supplement).

The identified statistical adjustment techniques were reviewed for relevance to the EU/EEA HIV surveillance data and discussed with the advisory group.

## Results

### Exploratory analysis

#### Missing data

In the case-based data analysed, 235,025 of 535,434 cases (43.9%) had complete information in all of the following key epidemiological variables: age, sex, transmission category, CD4^+^ count at diagnosis and migration status. These cases were defined as complete cases. Migration status was considered complete if one of the following variables was provided: country of birth, region of origin, country of nationality.

The low percentage of complete cases was mainly explained by CD4^+^ count, which was only included in European reporting as of 2008, as the variable with the highest proportion of missing values. The percentage of complete cases increased from 25.9% in the pre-2000 period to 56.8% between 2012 and 2014. This corresponded to the improvement in CD4^+^ count availability, which increased from 31.5% among those diagnosed before 2000 to 63.1% for the 2012 to 2014 period (Table 1). Some countries did not provide any CD4^+^ counts (Croatia, Germany, Hungary, Iceland, Norway, Poland and Sweden) and some countries were only able to report this variable to the EU/EEA dataset for more recent years (Estonia, Ireland, France, Slovakia, Lithuania, Malta, Denmark, Finland, Lichtenstein, Latvia, Belgium, Cyprus and Luxembourg). In these cases, CD4^+^ count is systematically missing either overall or for the earlier years.

**Table 1:** Availability of CD4^+^ count, transmission category, migrant status, age and sex in European HIV surveillance data over different diagnosis periods, EU/EEA countries (n = 31), 1984–2014^a^

Epidemiological variables	Diagnosis year										Overall	
	pre-2000		2000–04		2005–08		2009–11		2012–14			
	n = 135,957		n = 99,418		n = 111,586		n = 92,854		n = 95,619		n = 535,434	
	n	%	n	%	n	%	n	%	n	%	n	%
CD4^+^ count	42,801	31.5	49,359	49.6	52,926	47.4	56,277	60.6	60,329	63.1	261,692	48.9
Transmission category	121,293	89.2	87,797	88.3	94,010	84.2	77,030	83.0	78,156	81.7	458,286	85.6
Migrant status	89,671	66.0	84,23	84.7	94,198	84.4	79,548	85.7	81,993	85.7	429,649	80.2
Age	133,784	98.4	98,791	99.4	111,028	99.5	92,462	99.6	95,372	99.7	531,437	99.3
Sex	134,984	99.3	99,143	99.7	111,118	99.6	92,500	99.6	95,396	99.8	533,141	99.6
Complete records	35,158	25.9	45,165	45.4	48,865	43.8	51,545	55.5	54,292	56.8	235,025	43.9
Complete records (excluding CD4^+^ count)	83,867	61.7	78,465	78.9	86,719	77.7	72,379	77.9	73,476	76.8	394,906	73.8

EEA: European Economic Area; EU: European Union.

^a^ Case-based data were not available for all periods for Bulgaria, Estonia, France, Ireland, Italy and Spain.

Migration status could be determined for 87.8% of the cases diagnosed during all analysed time periods after 2000, compared with 66.0% for those diagnosed pre-2000. On the other hand, the proportion of individuals with known transmission category decreased slightly from 89.2% among those diagnosed pre-2000 to 81.7% for those diagnosed between 2012 and 2014. Age and sex were consistently reported in proportions higher than 98% during all periods (Table 1).

**Figure 1:**
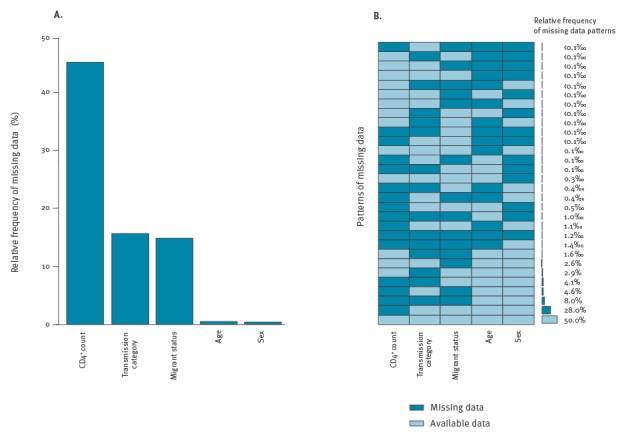
Frequency of missing data in key variables (A) and corresponding missing data patterns (B) in European HIV surveillance data, EU/EEA countries (n=31), 2000–2014^a^

EEA: European Economic Area; EU: European Union.

^a^ Case-based data were not available for all periods for Bulgaria, Estonia, France, Ireland, Italy and Spain.

The patterns of missing data were analysed for the cases diagnosed after 2000 (n = 399,477), given the higher relevance of the more recent estimates. Figure 1 shows the relative frequencies of missing data in each key variable. The panel A refers to total proportion of the missing values independently for each variable, while the panel B demonstrates how often the values of the examined variables were missing jointly in different combinations. There were 30 different patterns. Approximately 50% of the cases were complete in all key variables, which was the most common pattern (Figure 1, panel B). Further, for 28.0% of cases, CD4^+^ counts were missing but values of all the remaining key variables were available. The third row from the bottom in Figure 1, panel B shows that in 8% of the cases CD4^+^ counts, transmission category and migrant status is missing, but age and sex are available. Various other patterns with lower frequencies were also observed, representing 14% of the total number of cases.

**Figure 2:**
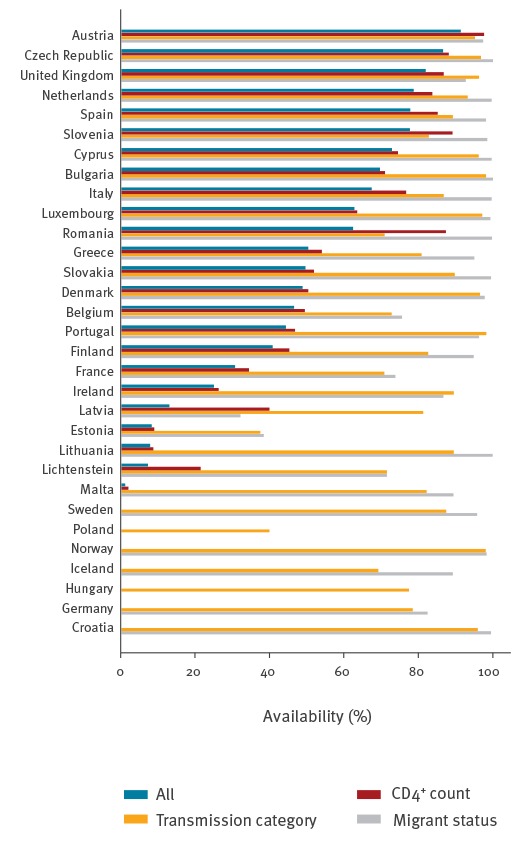
Proportion of European HIV surveillance records with simultaneous and per variable availability of CD4^+^ counts, transmission category and migrant status by reporting country, EU/EEA countries, 2000–2014

EEA: European Economic Area; EU: European Union.

Figure 2 shows the country-specific completeness of reporting for CD4^+^ count, transmission category and migrant status. The proportion of complete cases was highly variable across countries, ranging from 0 to almost 90%, and was mainly driven by the availability of CD4^+^ counts. Missing values in CD4^+^ cell count, transmission category and migrant status tended to occur simultaneously. Kendall’s τ ranged from 0.30 to 0.48 for the three possible pairwise associations of the missing value indicators of these variables, suggesting that the occurrence of missing values in these variables is not completely random.

Simultaneous availability is defined as availability of CD4^+^ counts, transmission category, migrant status, age and sex. For some countries the information presented does not cover the entire period, as the case-based data were only available for the following years: Bulgaria and Estonia (2007−14); Spain, France and Ireland (2003−14) and Italy (2010−14).

#### Reporting delay

We investigated the reporting delay between case diagnosis and notification at the national level. The delays were the longest for nine countries: Greece, France, Italy, Luxembourg, the Netherlands, Poland, Portugal, Sweden and the United Kingdom (UK). For 10 countries reporting delay was not calculated, either because the date of diagnosis was always equal to the date of notification (seven countries) or because the date of notification was not reported (three countries). In reporting to TESSy, countries include cases diagnosed during the submission year and notified up to a certain, country-specific date during the subsequent year. The number of quarters in the subsequent year for which data are still included is the minimal truncation time, which is applicable to cases diagnosed during the last quarter of the submission year. We considered that a delay is relevant in a country if more than 5% of cases are notified with a delay of two or more quarters in excess of the minimal truncation time. In 12 countries, the reporting delays were considered less relevant because 95% of cases were reported by the minimal truncation time (five countries) or within one quarter (three countries) or two quarters (four countries) in excess of the minimal truncation time. Although there was no direct association between the size of the country and the length of reporting delay, we note that the delay was relevant in all large EU/EEA countries, where it could be calculated, i.e. France, UK, Italy, Poland, and the other large countries, Germany and Spain, did not report data necessary for calculation of reporting delay.

**Figure 3:**
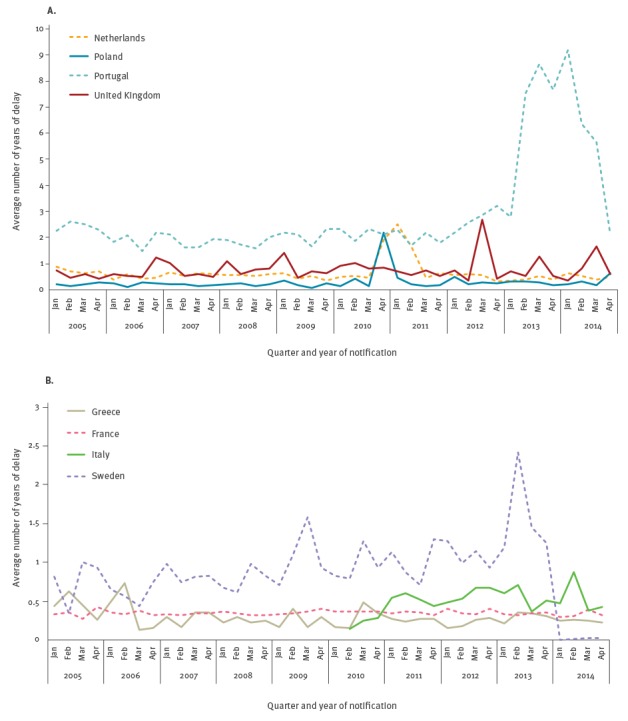
Average delay between HIV diagnosis and notification by the quarter of notification and reporting country, eight EU countries^a^, 2005–2014

EU: European Union; HIV: human immunodeficiency virus.

^a^ Greece, France, Italy, Netherlands, Poland, Portugal, Sweden, United Kingdom.

For eight of the nine countries with relevant reporting delay, the evolution of the average reporting delay over time is shown in Figure 3. Data from Luxembourg were not included in Figure 3 due to a low number of cases.

The average reporting delays show peaks in Portugal (2013–14), the Netherlands (2010–11) and Poland (2010). Furthermore, irregular patterns with occasional, less distinct peaks were noted in Italy and the UK.

Assuming that nearly all cases are reported within 5 years, we analysed cases diagnosed between 2001 and 2009 to look for patterns that may reasonably be expected to continue at later time periods. Data from Italy were excluded, as they were only available for 2010 – 2014.

For cases diagnosed in 2007–2009 the mean reporting delay ranged from 0.9 quarters in Greece to 5.6 quarters in Portugal. Significant changes over time were observed in all of the studied countries but Luxembourg, with decreasing tendency in all but Poland. Other important predictors of the delay distribution included transmission category and migrant status. Notably, the migration status was especially important in Luxemburg and Sweden (average delay among migrants was 5.8 and 3.3 quarters respectively, vs 1.9 and 1.0 quarters in native cases) (Table 2).

**Table 2:** Reporting delays for HIV cases by diagnosis date, transmission category and migration status, eight EU countries^a^, 2001–2009

Reporting delay (quarters)^b^		Reporting country							
		Greece	France	Luxembourg	The Netherlands	Poland	Portugal	Sweden	United Kingdom
**Date of diagnosis**									
2001–03	Mean	1.7	2.0	6.9	3.0	0.5	13.1	3.9	3.0
	IQR	0–1	1–2	0–6	1–4	0–1	1–21	0–1	0–3
2004–06	Mean	1.2	1.5	5.4	1.5	2.1	9.6	2.6	3.0
	IQR	0–1	0–2	0–2	0–1	0–1	1–16	0–1	0–3
2007–09	Mean	0.9	1.3	3.0	1.3	2.0	5.6	1.4	2.3
	IQR	0–1	1–2	0–1	0–1	0–1	0–7	0–1	1–3
p value (equality of median)		0.001	< 0.001	0.712	< 0.001	< 0.001	< 0.001	< 0.001	< 0.001
**Transmission category**									
Heterosexual	Mean	0.9	1.4	4.2	1.7	1.0	9.5	2.7	2.8
	IQR	0–1	0–2	0–1	0–2	0–1	1–15	0–1	0–3
Injecting drug use	Mean	2.0	1.6	6.8	3.4	1.0	10.2	1.5	2.7
	IQR	0–1	1–2	0–1	0–4	0–1	1–15	0–1	0–3
Men who have sex with men	Mean	1.6	1.3	5.4	1.7	1.3	9.3	2.4	2.6
	IQR	0–1	0–1	0–8	0–1	0–1	1–14	0–1	0–2
Other	Mean	1.2	2.2	10.8	6.8	1.3	12.8	7.3	3.3
	IQR	0–1	1–2	0–21.5	1–10	0–1	1–22	0–12	1–3
Unknown	Mean	0.6	1.7	8.2	2.2	2.0	10.5	1.9	3.9
	IQR	0–0	1–2	0–14	0–2	0–1	1–17	0–1	1–4
p value (equality of median)		< 0.001	< 0.001	0.138	< 0.001	< 0.001	< 0.001	< 0.001	< 0.001
**Migration status**									
Originating from the reporting country	Mean	1.3	1.4	1.9	1.4	NA^c^	9.4	1.0	2.6
	IQR	0–1	0–1	0–0	0–1	NA	1–14	0–1	0–2
Originating from abroad	Mean	1.0	1.4	5.8	2.4	NA	9.0	3.3	2.7
	IQR	0–1	0–2	0–6	0–2	NA	1–13	0–1	0–3
p value (equality of median)		0.06	0.024	0.001	< 0.001	NA	0.518	< 0.001	0.001
**Number of inhabitants (in millions)^d^**		**11.1**	**64.4**	**0.5**	**16.5**	**38.1**	**10.6**	**9.3**	**62.0**

IQR: interquartile range; NA: not available.

^a^ Greece, France, Luxembourg, Netherlands, Poland, Portugal, Sweden, United Kingdom.

^b^ Medians not presented as they predominantly equal 0.

^c^ Migration status missing for all cases.

^d^ According to Eurostat, 2009 [33].

### Suggested adjustment techniques

Based on our literature search we selected 28 articles (including seven reviews) and five textbook passages discussing the missing values, as well as 27 articles (including two reviews) on reporting delay corrections, which were relevant to the EU/EEA HIV surveillance data (Supplement).

#### Missing data

When accounting for missingness, one of the following mechanisms regarding the missing data needs to be considered [3]:

data missing completely at random (MCAR), if the probability that a value is missing neither depends on the value itself nor on any other factors including observable covariates;
data missing at random (MAR), if the probability that a value is missing does not depend on the value itself but may depend on other covariates; and
data missing not at random (MNAR), if the probability that a value is missing may in fact depend on the value, which is not observed, e.g. transmission category is not recorded as sex between men due to possible stigma.

It is not possible to discriminate between MAR and MNAR based on the observed data alone. Usually, external information is required, for example an expert opinion regarding the details of the data collection process. On the other hand, the MCAR mechanism is rarely encountered and the analysis typically begins with an assumption of MAR. A tendency towards simultaneous incompleteness of several variables, as was the case in the HIV surveillance data (Figure 2), indicates that the data are not MCAR.

It is also useful to check if the data follow a monotone pattern of missing values. In this pattern, the incomplete variables can be ordered so that if the value of the first variable is missing then the value of the second variable is missing, as well as the values of all the following variables. Further, regardless of the first variable, if the value of the second variable is missing, then the value of the third one and all the following variables are also missing and so on. However, as shown in the patterns of missing data in the EU/EEA HIV surveillance data (Figure 1B), monotonicity does not hold.

**Figure 4:**
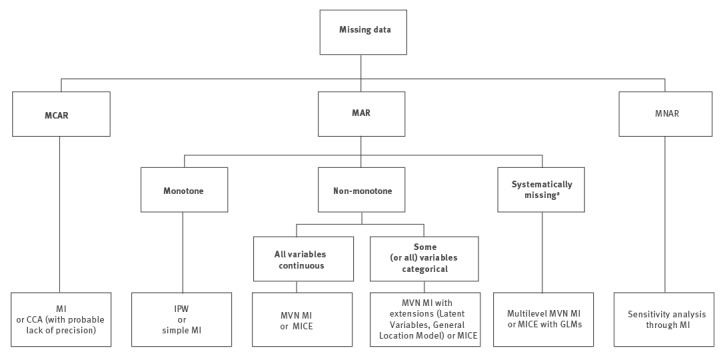
Recommended treatment of missing data in EU/EEA HIV surveillance data

CCA: complete case analysis; GLM: generalised linear models; IPW: inverse probability weighting; MAR: missing at random; MCAR: missing completely at random; MI: multiple imputation; MICE: multiple imputation by chained equations; MNAR: missing not at random; MVN: multivariate normal.

^a^ Systematically missing refers to a variable, for which all values are missing, e.g. it is not collected at all in a country.

Typically, the missing value adjustments rely solely on the analysed dataset and do not require additional data. The simpler techniques, e.g. complete/available case analysis, mean imputation and last observation carried forward, are prone to various problems, including loss of power, bias, dilution of associations and underestimation of uncertainty, and should be avoided [4]. Moreover, some theoretically grounded techniques, such as inverse probability weighting [11], require a monotone pattern of missing values (Figure 4). Even though the observed pattern of missing values in HIV surveillance data at the EU/EEA level is not monotone (Figure 1B), this may be the case for national data.

The method of multiple imputations (MI), first introduced by Rubin in 1987 [12], has the potential to overcome the issues outlined above when MCAR or MAR mechanisms are valid. The MI method involves filling each of the missing values with values randomly sampled from an appropriate distribution. The imputation is performed *M* times, typically 5– 10, and in effect, we obtain *M* so-called pseudo-complete datasets [3,4,12]. The model of interest, also called substantive model, can be fitted to each of the imputed datasets in order to estimate the parameter of interest and its variance *M* times. These can be combined using Rubin’s rules [3] to obtain an overall, i.e. average over *M* estimator and its associated variance.

Drawing samples from the distribution of the missing data, conditioning on the observed ones to fill in missing values, is relatively simple when there is only one partially observed variable or for monotone pattern, as it involves estimation of an appropriate regression model [12]. For non-monotone missing value patterns, the main approaches of MI are based on joint modelling, e.g. multivariate normal model or full conditional specification, i.e. multiple imputations by chained equations (MICE) (Figure 4).

The multivariate normal imputation model relies on the assumption that the joint distribution of all variables under consideration is in fact the multivariate normal. For a smaller proportion of missing values, MI may produce robust results even if this assumption is not met, as the imputation model is only applied to the missing part of the data [13]. However, in case of a high proportion of missing values, under specific violations of multivariate normality and depending on the focus of the analysis, the results may be biased [14,15].

More generally, even though there are no clear guidelines on acceptable levels of missing values, it is expected that any violation of a model’s assumptions will have more pronounced consequences with high proportions of missing data. In the EU/EEA HIV surveillance data, the level of missing values in most of the key covariates is below 20% (Figure 1), with the exception of CD4^+^ count. In countries with sparse CD4^+^ data, transformation of CD4^+^ count to a normal distribution should be carefully considered. It would also be useful to consider increasing the number of imputations beyond the typically used number of 5–10, as the estimates can be inaccurate otherwise [16].

If the data contain a mixture of continuous and categorical variables, multivariate normal MI can be extended to the latent normal or general location models [17]. Alternatively, multiple imputations can be performed with the full conditional specification method (MICE) (Figure 4). With the MICE method, separate specific models are constructed for each of the variables to be imputed, depending on the type of these variables. These univariate models are fitted iteratively for each partially observed variable using both observed and previously imputed data of the remaining variables until the procedure converges. Then, a pseudo-complete dataset is generated and the whole procedure is repeated *M* times. MICE offers a flexible way of specifying the imputation models. However, the risk of this procedure is that the specified models may be incompatible, i.e. no joint model for which the conditional models would be described by the proposed univariate models exists [18,19].

Both the joint modelling and the full conditional specification approaches described above can be extended to datasets combining data from different studies or, as is the case with EU/EEA HIV surveillance data, different national surveillance systems [20,21]. The approach proposed by Quartagno et al. [20], which is based on multilevel multiple imputation and incorporating the idea of random covariances, and the approach of Jolani et al. [21], which applies generalised linear mixed models within the MICE framework, would also allow for imputation of covariates in national data in the countries where these covariates are completely missing, using other countries’ data in the EU/EEA surveillance dataset.

Importantly, all MI methods rely on the assumption that data are MAR. Although this is a plausible assumption, and rich imputation models increase the likelihood of satisfying it [22], one needs to assess the extent to which the data support the analysis conclusions under a range of scenarios of violations of the MAR assumption. Sensitivity analyses on the plausibility of the MAR assumption can be performed either within a pattern mixture model framework or in a simpler approximate method based on the idea of up-weighting imputations that are more likely under an MNAR mechanism [23].

#### Reporting delay

Reporting is often considered in discrete time frameworks, e.g. years, quarters, months, and, typically, the same time units are used to describe the reporting delay. With EU/EEA HIV surveillance data, the conventional time unit is one quarter. The majority of modern adjustment techniques rely on estimation of the delay distribution independently of the diagnosis rate [24-26]. Once the estimate for the delay distribution is obtained, it is used to estimate the proportion of cases already reported, given the diagnosis date and the cut-off date for reporting, i.e. truncation time. Estimating this proportion allows for adjusting the diagnosis rate.

The reporting delay distribution can be estimated in a non-parametric way using a multinomial model, assuming there is a maximum delay [5], or using the reverse time transform and estimating the survivorship function with left-truncated data [27]. In practice, both the confidence intervals and the point estimates for the delay probabilities are equivalent for the two approaches. Alternatively, missing data techniques, as discussed above, could be applied. Using these methods, the counts of the cases, which will be reported with delay, are treated as missing and imputed [28].

A fully non-parametric estimation allowing arbitrary trends of the delay distribution over time is not possible. Therefore, additional assumptions have to be made: that the delay distribution is not changing over time, the so-called quasi-stationarity assumption, or that it is stable in time intervals, or that the trend can be described by a regression model [24,26,29]. Regression models also offer a convenient way to test the quasi-stationarity assumption [29] and possibly simplify the estimation if this assumption is reasonable.

The overriding issue in estimation of the delay distribution is the right truncation. However, double truncation, i.e. left and right truncation, may apply to countries with long reporting delays where surveillance was implemented with delay and data on earlier diagnoses may not be available. The methods for non-parametric estimation in such instances are more complex and may not be available in standard statistical packages [30,31]. An interesting alternative to fully non-parametric models is to model the hazard function parametrically, choosing appropriately flexible functions such as splines [6]. Finally, some methods have already been developed to account for random variation of the reporting delay, e.g. a multinomial model with random effects [5].

## Discussion

The exploration of data quality issues in the EU/EEA HIV surveillance data showed that both missing data and reporting delay were present to varying degrees across countries and over time. Addressing these issues requires the application of proper adjustment techniques and clear understanding of national data in order to optimise the validity of estimates derived from these data.

Considering the complex nature of missing data in the EU/EEA HIV surveillance data, the lack of monotonicity in the missing data patterns, the high likelihood of MAR or even MNAR mechanisms and the variety of potential analyses of interest, multiple imputation methods seem to provide an optimal framework for inference [3,4]. Joint modelling and full conditional specification [18,19] approaches are flexible and mature enough to accommodate even complex analyses. Their potential for multilevel extensions [17,20,21] might prove very useful when combining evidence across EU/EEA countries, especially for countries with systematically missing CD4^+^ counts. Moreover, MI methods offer a very flexible framework for sensitivity analyses regarding the possibility of non-random missingness [23].

The reporting delays encountered in the EU/EEA HIV surveillance data were noteworthy only for a fraction of the countries. However, among these countries we noted marked differences with respect to the average delay, observable trends over time and differences between subpopulations. Moreover, in several countries the average reporting delays are much higher for selected notification quarters. These increases are possibly related to control activities in surveillance, ‘cleaning events’ during which previously unreported cases are discovered and notified with delay. In other countries irregular patterns are observed, which could be related to random situations that may occasionally delay the notification process, including at local levels. Certainly though, in particular situations, other mechanisms may result in similar data patterns.

The reporting delay correction method introduced in the EuroHIV project, which coordinated HIV/AIDS surveillance in the WHO European Region between 1984 and 2007, and applied also by the ECDC, assumes that the delay distribution remains stationary [8,9]. This is not the case in all countries. Appropriate country-specific regression models, accounting for right truncation of data, which allow to include the trends in reporting delays along with other predictors, should therefore be considered [8,24,27,29]. Two particular issues that may require special treatment are (i) the long tails of the delay distributions, which are problematic when the data are not available from the start of an epidemic (e.g. Italy) and (ii) extra-variability because of fluctuations of reporting systems (e.g. UK, Italy) or control events (e.g. Portugal, Poland, the Netherlands). The former can be dealt with by using the methods for doubly truncated data [6,30,31]. The random fluctuations of the surveillance systems may be approached via random effect models [5]. However, the problem of the cleaning events may still pose a challenge.

There is a clear need both to improve the quality of surveillance data at the systems level and to employ statistical procedures to reliably adjust estimates and quantify their uncertainty. At the time of consultation in 2015, only three countries (France, Spain and the UK) reported routinely using statistical corrections in line with those mentioned in this study [9,32]. This may suggest a lack of capacity in some countries to apply these methods. The wider use of the adjustments would not only improve the national estimates, but also contribute to higher comparability and harmonisation of data across EU/EEA. We therefore recommend applying these adjustments for national estimates, preceded, if needed, by capacity building at the national level. Usage of the adjustments requires careful considerations because of the complexity of the data and their heterogeneity across countries and over time. Importantly, some variables are collected in national surveillance systems that are not later on forwarded to the EU/EEA surveillance system even though these variables may be essential to effectively correct for the missing data and reporting delay.

The next steps, in terms of refining and optimising the identified techniques, include the application and validation of several generic models, taking into account particular covariates collected in the surveillance systems to guide future use of these techniques at national or EU/EEA level.

Furthermore, as data incompleteness and delay in reporting are by no means unique to HIV surveillance, the adjustment techniques discussed here may also be applicable to other surveillance systems with similar data quality problems.

## Notes

**Members of the ECDC HIV/AIDS Surveillance Network:** Austria: Daniela Schmid; Belgium: Andre Sasse, Dominique Van Beckhoven; Bulgaria: Tonka Varleva; Croatia: Tatjana Nemeth Blazic; Cyprus: Linos Hadjihannas, Maria Koliou; Czech Republic: Marek Maly; Denmark: Susan Cowan; Estonia: Kristi Rüütel; Finland: Kirsi Liitsola, Mika Salminen; France: Françoise Cazein, Josiane Pillonel, Florence Lot; Germany: Barbara Gunsenheimer-Bartmeyer; Greece: Georgios Nikolopoulos, Dimitra Paraskeva; Hungary: Maria Dudas; Iceland: Haraldur Briem, Gudrun Sigmundsdottir; Ireland: Derval Igoe, Kate O’Donnell, Darina O’Flanagan; Italy: Barbara Suligoi; Latvia: Šarlote Konova; Liechtenstein: Sabine Erne; Lithuania: Irma Čaplinskienė; Luxembourg: Aurelie Fischer Jean-Claude Schmit; Malta: Jackie Maistre Melillo, Tanya Melillo; the Netherlands: Eline Op de Coul, Ard van Sighem; Norway: Hans Blystad; Poland: Magdalena Rosinska; Portugal: Isabel Aldir, Helena Cortes Martins; Romania: Mariana Mardarescu; Slovakia: Peter Truska; Slovenia: Irena Klavs; Spain: Asuncion Diaz; Sweden: Maria Axelsson; United Kingdom: Valerie Delpech.
